# Comparative effectiveness of neuroendoscopic surgery and stereotactic aspiration for brain hemorrhage

**DOI:** 10.3389/fsurg.2026.1782293

**Published:** 2026-04-01

**Authors:** Hazrat Jalal, Huikai Zhang, Long Zhou, Zhiyang Li, Jiajun Wei, Shenqi Zhang, Qiang Cai

**Affiliations:** Department of Neurosurgery, Renmin Hospital of Wuhan University, Wuhan, Hubei, China

**Keywords:** functional independence, intraparenchymal hemorrhage, neuroendoscopic surgery, neurological improvement, stereotactic aspiration

## Abstract

**Background:**

Intraparenchymal hemorrhage (IPH) involving the cerebrum, cerebellum, and brainstem is a critical condition with high mortality. While minimally invasive surgical techniques are widely utilized, the comparative effectiveness of neuroendoscopic surgery (NS) vs. stereotactic aspiration (SA) across different anatomical locations remains underexplored. This study aims to retrospectively compare the effectiveness and safety of NS and SA in a cohort encompassing different IPH locations.

**Methods:**

A single-center retrospective analysis was conducted on 199 patients with IPH (NS: *n* = 97; SA: *n* = 102) treated between 2019 and 2023. The primary outcome was the median hematoma reduction rate (%) and included acute neurological improvement [change in Glasgow Coma Scale (GCS) at 24 h postoperatively]. Secondary outcome: functional independence [modified Rankin Scale (mRS) 0–3] at discharge. Multivariate logistic regression adjusted for baseline imbalances in age and hypertension. Radiologic evacuation and neurological change were evaluated as early surrogate endpoints and do not directly measure long-term functional recovery.

**Results:**

Overall, NS demonstrated a significantly higher median hematoma reduction rate compared to SA (92.90% vs. 22.20%, *p* < 0.001) and greater acute neurological improvement (median ΔGCS 4.0 vs. 0.5 points, *p* < 0.001). These trends were consistently observed across deep-seated, lobar, cerebellar, and brainstem subgroups (all *p* < 0.05). Functional independence at discharge was achieved by 27.8% in the NS group vs. 15.7% in the SA group (*p* = 0.040). Furthermore, NS was associated with significantly lower symptomatic rebleeding (7.2% vs. 24.5%, *p* < 0.001) and 30-day mortality (9.3% vs. 22.5%, *p* = 0.012).

**Conclusion:**

This retrospective analysis suggests that NS is associated with higher evacuation efficiency and more pronounced early neurological recovery across various IPH locations compared to SA. While prospective validation is required to confirm long-term functional trajectories, these findings highlight the potential advantages of direct visualization and active hemostasis in managing IPH across different anatomical locations.

## 1 Introduction

Intraparenchymal hemorrhage (IPH) represents a critical neurosurgical emergency, accounting for approximately 10%–15% of all stroke cases worldwide (1). Recent epidemiological data suggest that in certain regions, spontaneous IPH can account for up to 25%–27% of all stroke subtypes, with an annual global incidence affecting over 5 million people (2, 3). Spontaneous IPH is associated with profound morbidity and a 30-day mortality rate of approximately 40%, which can escalate to 54% within the first year (2). These hemorrhages are traditionally categorized by their anatomical distribution, including lobar, thalamic, or basal ganglia locations, with each site posing distinct surgical and prognostic challenges. While deep IPH (basal ganglia/thalamus) is frequently associated with hypertensive small vessel disease, lobar (subcortical) IPH is often linked to cerebral amyloid angiopathy; both locations represent the vast majority of clinical cases and require tailored surgical strategies (3, 4).

The anatomical location of a hematoma is a primary determinant of the clinical trajectory. Cerebellar hemorrhage represents 5%–13% of spontaneous IPH and frequently results in obstructive hydrocephalus or brainstem compression, with reported mortality rates ranging from 20% to 75% (5, 6). Brainstem hemorrhage, while less frequent, carries a high fatality risk due to the potential disruption of vital regulatory centers; primary brainstem hemorrhage, often linked to chronic hypertension, is particularly severe. Mortality rates for brainstem bleeds can reach 90% when volumes exceed 5 mL and nearly 100% for volumes larger than 10 mL (7). Consequently, IPH involving the cerebrum, cerebellum, and brainstem constitutes a spectrum of life-threatening conditions that often require surgical intervention to mitigate secondary brain injury (3).

Minimally invasive surgical (MIS) techniques have emerged as promising alternatives to traditional craniotomy. NS offers the potential advantage of direct visualization (8), enabling efficient clot evacuation and meticulous hemostasis. Contemporary studies have demonstrated that NS can achieve superior hematoma evacuation rates, often exceeding 89%–90%, which correlates with significant improvements in Glasgow Coma Scale (GCS) scores at discharge compared to traditional approaches (2, 4). Notably, subcortical (lobar) hemorrhages may be particularly amenable to NS, with evacuation efficiencies reaching as high as 92.7% (4). Conversely, SA utilizes image-guided catheter placement to aspirate liquefied hematomas, often augmented by thrombolytic therapy. SA is considered a viable option for deep-seated or technically demanding locations and remains a valuable tool for high-risk patients who may not tolerate prolonged anesthesia (9–11).

Despite the growth of MIS options, significant gaps remain in the comparative literature. Most clinical investigations have focused on supratentorial hemorrhages, leaving a lack of data for cerebellar and brainstem locations where anatomical complexity introduces unique surgical risks (12, 13). Furthermore, while MIS has demonstrated general benefits over conventional craniotomy in trials such as STICH and MISTIE III (14, 15), few studies have provided direct comparisons between NS and SA across different anatomical locations within a single institutional framework (16, 17). The influence of specific hematoma locations on the relative effectiveness of these two MIS techniques remains underexplored.

To address these gaps, we retrospectively compared the effectiveness and safety of NS vs. SA across different IPH anatomical locations to provide clinically relevant insights for anatomically guided surgical decision making.

## 2 Materials and methods

### 2.1 Study design and patient cohort

This single-center retrospective study assessed the effectiveness of NS and SA in a cohort of 199 Chinese patients with IPH hospitalized in the Department of Neurosurgery, Renmin Hospital of Wuhan University, Wuhan, China. Institutional records from 2019 to 2023 identified 199 eligible patients (NS: 97; SA: 102). Hemorrhage was confirmed on initial Computed Tomography (CT) scans upon hospitalization and hematoma volumes were measured via 3D Slicer software (http://www.slicer.org). The choice of surgical modality (NS vs. SA) was determined by the attending neurosurgical team based on a comprehensive assessment of the patient's clinical presentation (including GCS score and hematoma volume), anatomical location of the hemorrhage, and the specific expertise of the operating surgeon. Additionally, discussions regarding the risks and benefits of each technique were held with the patients' families, and their preferences were incorporated into the final surgical plan. All lead surgeons involved in this cohort possessed over 10 years of specialized expertise in both NS and SA techniques, ensuring technical proficiency across both treatment arms.

### 2.2 Eligibility criteria

#### 2.2.1 Inclusion criteria

Patients were selected for this study based on a strict set of pre-defined eligibility criteria to ensure cohort homogeneity and data integrity. Inclusion required a diagnosis of an acute, severe neurological condition resulting from primary spontaneous IPH, involving deep-seated (Basal Ganglia/Thalamus), lobar regions, primary intraventricular hemorrhage (IVH), cerebellum, and brainstem, as definitively confirmed by non-contrast computed tomography (CT). Acute presentation was defined as a time from symptom onset to surgical intervention of less than 48 h. Furthermore, eligible patients must have undergone a minimally invasive surgical intervention, specifically NS or SA, performed within the acute phase of the hemorrhage. Finally, the availability of a comprehensive and complete medical record encompassing all clinical, radiological, and follow-up variables required for the study throughout the specified institutional period was mandatory for inclusion.

#### 2.2.2 Exclusion criteria

To ensure a focused analysis of primary IPH, we excluded patients with secondary etiologies (AVMs, aneurysms, tumors, or ischemic transformation) and traumatic injuries. To maintain a strictly minimally invasive cohort, patients managed conservatively or via open craniotomy were also excluded. Finally, patients were removed due to acute secondary injuries, incomplete clinical/radiological records, or loss to follow-up and center transfers.

### 2.3 Surgical procedures

All procedures were performed according to institutional protocols under general anesthesia in a standardized operating room environment.

#### 2.3.1 Stereotactic aspiration (SA)

For supratentorial cases, the frontal puncture point was typically marked 2.5 cm lateral to the midline and 11 cm above the eyebrow root. For infratentorial cases, the puncture point was marked in the suboccipital region, typically 3 cm lateral to the midline and inferior to the transverse sinus. Using image guidance, a burr hole was created, and the dura mater was incised in a cruciate fashion. A brain cannula or biopsy needle was inserted parallel to the sagittal plane to the center of the hematoma. The initial liquid component was manually aspirated using a 10 mL syringe until significant resistance was met. A No. 12 flexible drainage catheter was then inserted along the established trajectory and secured to the scalp.

Postoperatively, the hematoma was continuously liquefied through the administration of a fibrinolytic agent (20,000–40,000 U of urokinase in saline solution) via the catheter over the following 2–4 days. Routine CT scans were performed at 24 and 72 h postoperatively to monitor progress and hematoma stability. Catheters were removed following the 72-hour CT scan once a stable residual volume was confirmed.

#### 2.3.2 Neuroendoscopic surgery

The surgical approach was tailored to the anatomical location. For supratentorial deep hemorrhages on the dominant side, a transcortical corridor through the middle temporal gyrus (MTG) was used; for other locations, the shortest-distance corridor was selected. The procedure began with a 4–5 cm linear scalp incision and a small circular craniotomy (2.5 cm diameter). Following a cruciate dural incision, a transparent sheath was carefully inserted. A Karl Storz high-definition endoscope was introduced through the sheath, enabling direct visualization and meticulous evacuation of the hematoma while achieving active hemostasis of bleeding points in the hematoma wall.

pRegarding patient positioning, supratentorial cases were performed in the supine position. For cerebellar hemorrhages, patients were positioned in a modified supine posture, with the head rotated 60°–90° toward the contralateral side and the neck slightly flexed to expose the suboccipital region adequately. In complex cases complicated by late-stage herniation or obstructive hydrocephalus, a frontal external ventricular drainage (EVD) was established before the primary evacuation to facilitate rapid reduction of intracranial pressure (ICP).

### 2.4 Postoperative management and outcome measurement

Following hematoma evacuation, patients were managed in the neurosurgical intensive care unit. Care focused on strict control of postoperative systolic blood pressure, maintained below 160 mmHg, and avoidance of excessive fluid administration.

#### 2.4.1 Radiological assessment

**Figure 1:**
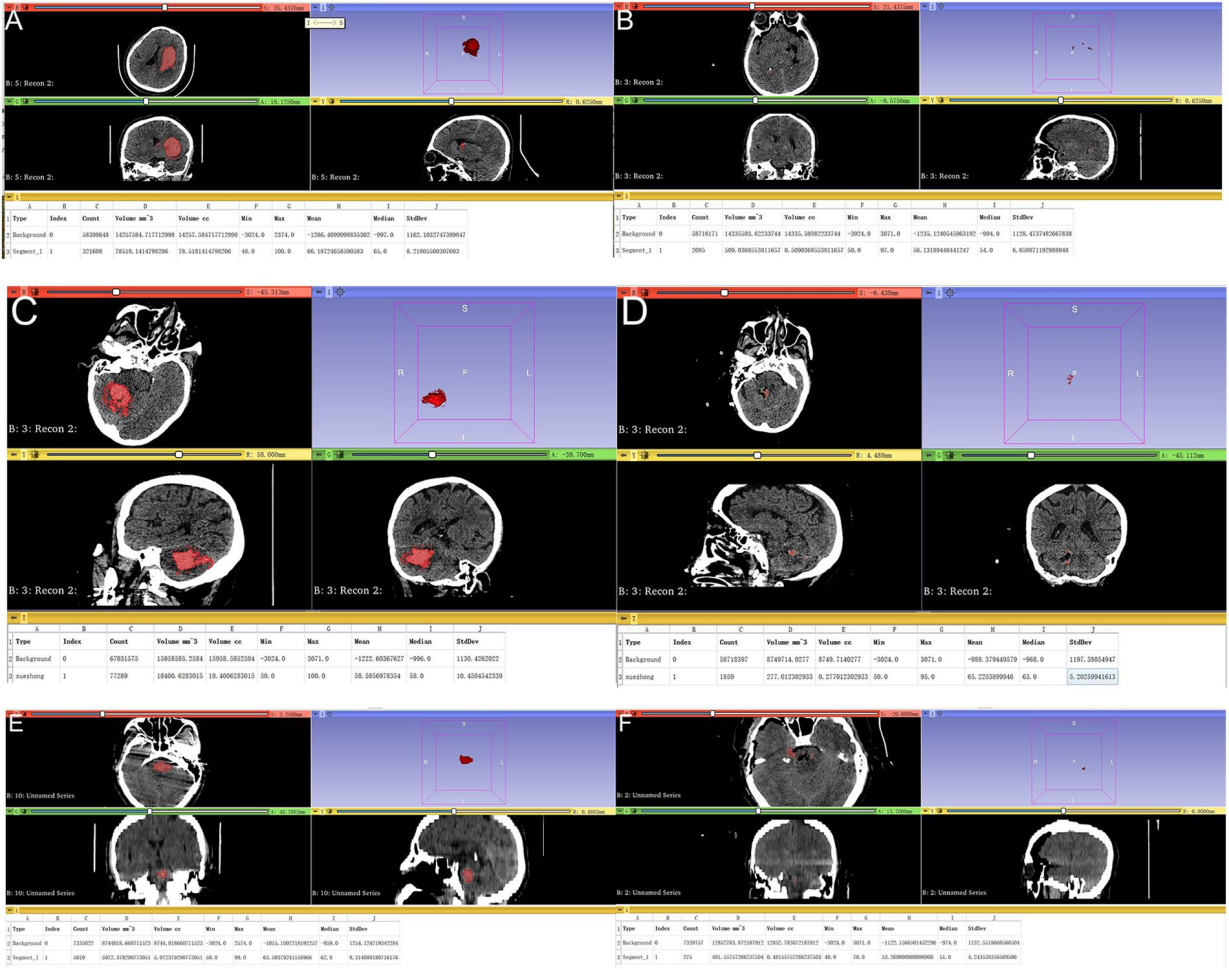
**(A,B)** Comparative pre- and postoperative CT images of a cerebral hematoma. CT images of a patient with a cerebral hematoma. The preoperative image **(A)** shows a volume of 78.51 mm^3^, which was successfully reduced to a minimal postoperative volume of 0.50 mm^3^ **(B)**, resulting in a 99.36% evacuation rate. **(C,D)** Comparative pre- and postoperative CT images of a cerebellar hematoma. This figure displays the CT scans of a patient with a cerebellar hematoma. The preoperative image **(C)** shows an initial volume of 18.40 mm^3^, which was reduced to a postoperative volume of 0.27 mm^3^ **(D)**, demonstrating a high evacuation rate of 98.53%. **(E,F)** Comparative pre- and postoperative CT images of a brainstem hematoma. The CT scans in this figure illustrate the change in a brainstem hematoma. The preoperative image **(E)** highlights an initial volume of 5.97 mm^3^, which was reduced to a postoperative volume of 0.48 mm^3^ **(F)**, achieving a notable evacuation rate of 91.96%.

To assess surgical outcomes, all patients underwent a postoperative CT scan within 24 h. Residual hematoma volumes for cerebral, cerebellar, and brainstem locations were precisely measured via 3D Slicer software, as illustrated in Figures 1A–F, respectively. The hematoma evacuation rate was calculated using the following formula:

$$\mathrm{Evacuation}\;\mathrm{Rate}=\frac{\mathrm{Preoperative}\;\mathrm{Volume}-\mathrm{Postoperative}\;\mathrm{Volume}}{\mathrm{Preoperative}\;\mathrm{Volume}}\times 100\text{\%}$$ (1)

#### 2.4.2 Outcome measures

The study assessed clinical and radiological outcomes through a hierarchical approach. The primary outcomes were the median hematoma reduction rate (%) and acute neurological improvement. The hematoma reduction rate was calculated by comparing the baseline hematoma volume with the 24-hour postoperative CT volume. Acute neurological improvement was defined as the change in Glasgow Coma Scale (GCS) scores from admission to 24 h postoperatively. The secondary outcome was functional independence at the time of hospital discharge, defined as a modified Rankin Scale (mRS) score of 0–3.

### 2.5 Statistical analysis

Statistical analyses were performed via the R programming language. Data normality was formally assessed using the Shapiro–Wilk test. Given the significant deviations from a normal distribution (*p* < 0.001), nonparametric methods were utilized throughout. Inter-group comparisons for continuous variables were conducted using the Wilcoxon rank sum (Mann–Whitney *U*) test and reported as medians with interquartile ranges (IQR). Categorical data were compared using Fisher's exact test.

To address baseline imbalances, specifically age and hypertension, and to control for initial clinical severity, a multivariate logistic regression model using Firth's penalized likelihood was employed to identify independent predictors of functional independence (mRS 0–3). The model included surgical group, age, hypertension, pre-operative GCS, and hematoma volume. Significance was defined as a two-tailed *p* < 0.05.

## 3 Results

### 3.1 Baseline characteristics and cohort balance

**Figure 2:**
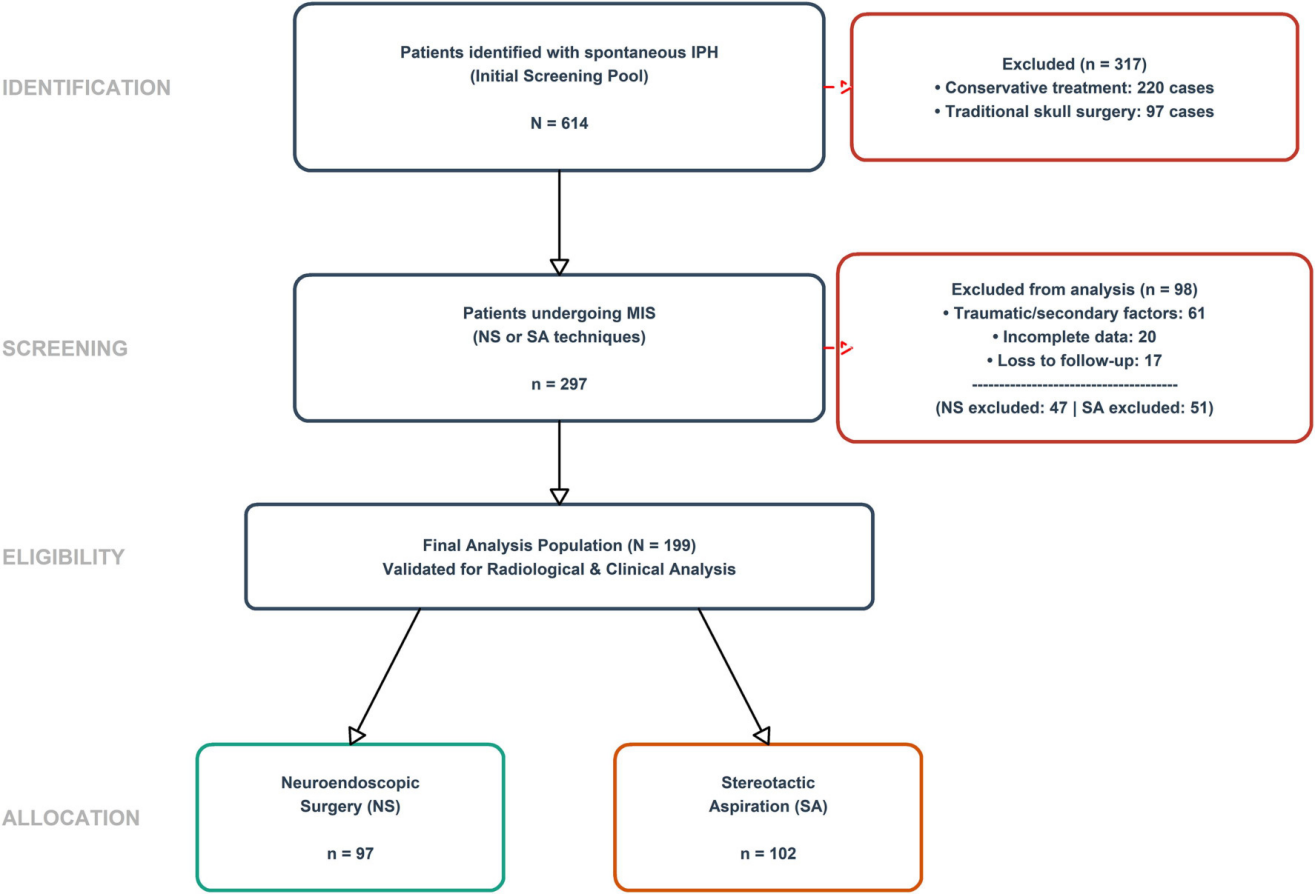
STROBE flow diagram of the study recruitment and selection process. From an initial pool of 614 patients identified with spontaneous intracranial hemorrhage, 317 were excluded for conservative or traditional treatment. Among the 297 patients who underwent minimally invasive surgery, 98 were further excluded due to traumatic factors, incomplete data, or loss to follow-up. The final analysis included 199 patients, allocated to the NS (*n* = 97) and SA (*n* = 102) groups. STROBE: Strengthening the Reporting of Observational Studies in Epidemiology.

A total of 199 patients were included in the final analysis (NS: *n* = 97; SA: *n* = 102) as illustrated in the study STROBE flow Figure 2. The anatomical distribution of IPH was statistically comparable across the cohorts (*p* = 0.602).

As shown in Table 1, the groups were balanced regarding sex (*p* = 0.880), pre-operative neurological severity (median GCS: 8.00 vs. 7.00; *p* = 0.062), pre-operative hematoma volume (median: 29.90 vs. 33.60 mL; *p* = 0.097). The prevalence of diabetes mellitus was also similar between groups (8.8% vs. 6.2%; *p* = 0.409). The median time from symptom onset to surgery was comparable between groups (NS: 11.80 vs. SA: 15.10 h; *p* = 0.061). However, significant baseline imbalances were observed in age (median 57.5 vs. 56.0 years; *p* = 0.017) and in hypertension prevalence (59.8% vs. 34.0%; *p* < 0.001). These imbalances were addressed through multivariate adjustment in the final analysis. Anatomical distribution was comparable across cohorts (*p* = 0.602; Table 1).

**Table 1:** Baseline clinical and radiological characteristics.

Characteristic	SA group (*n* = 102)	NS group (*n* = 97)	*p* value
Age (years), median [IQR]	57.5 [51.0, 65.0]	56.0 [46.0, 61.0]	0.017*
Sex (male), *n* (%)	68 (66.7%)	66 (68.0%)	0.880
Pre-op GCS, median [IQR]	8.00 [5.00, 10.00]	7.00 [4.00, 10.00]	0.062
Pre-op volume (mL), median [IQR]	29.90 [19.1, 58.3]	33.60 [24.0, 58.0]	0.097
Hemorrhage location, *n* (%)			0.602
Basal ganglia	51 (48.1%)	46 (47.4%)	
Thalamus	14 (13.2%)	9 (9.3%)	
Brainstem	15 (14.7%)	15 (15.5%)	
Cerebellum	10 (9.8%)	10 (10.3%)	
Intraventricular (IVH)	6 (5.9%)	5 (5.2%)	
Lobar	6 (5.9%)	12 (12.4%)	
Onset to surgery (h), med [IQR]	15.10 [8.80, 41.00]	11.80 [6.0, 20.50]	0.061
Comorbidities, *n* (%)			
Hypertension (HTN)	61 (59.8%)	33 (34.0%)	<0.001*
Diabetes mellitus (DM)	9 (8.8%)	6 (6.2%)	0.409

Continuous variables are reported as median [IQR] unless specified. Baseline imbalances in HTN were accounted for in the multivariate logistic regression analysis.

* Statistically significant (*p* < 0.05).

### 3.2 Radiological and neurological outcomes

**Figure 3:**
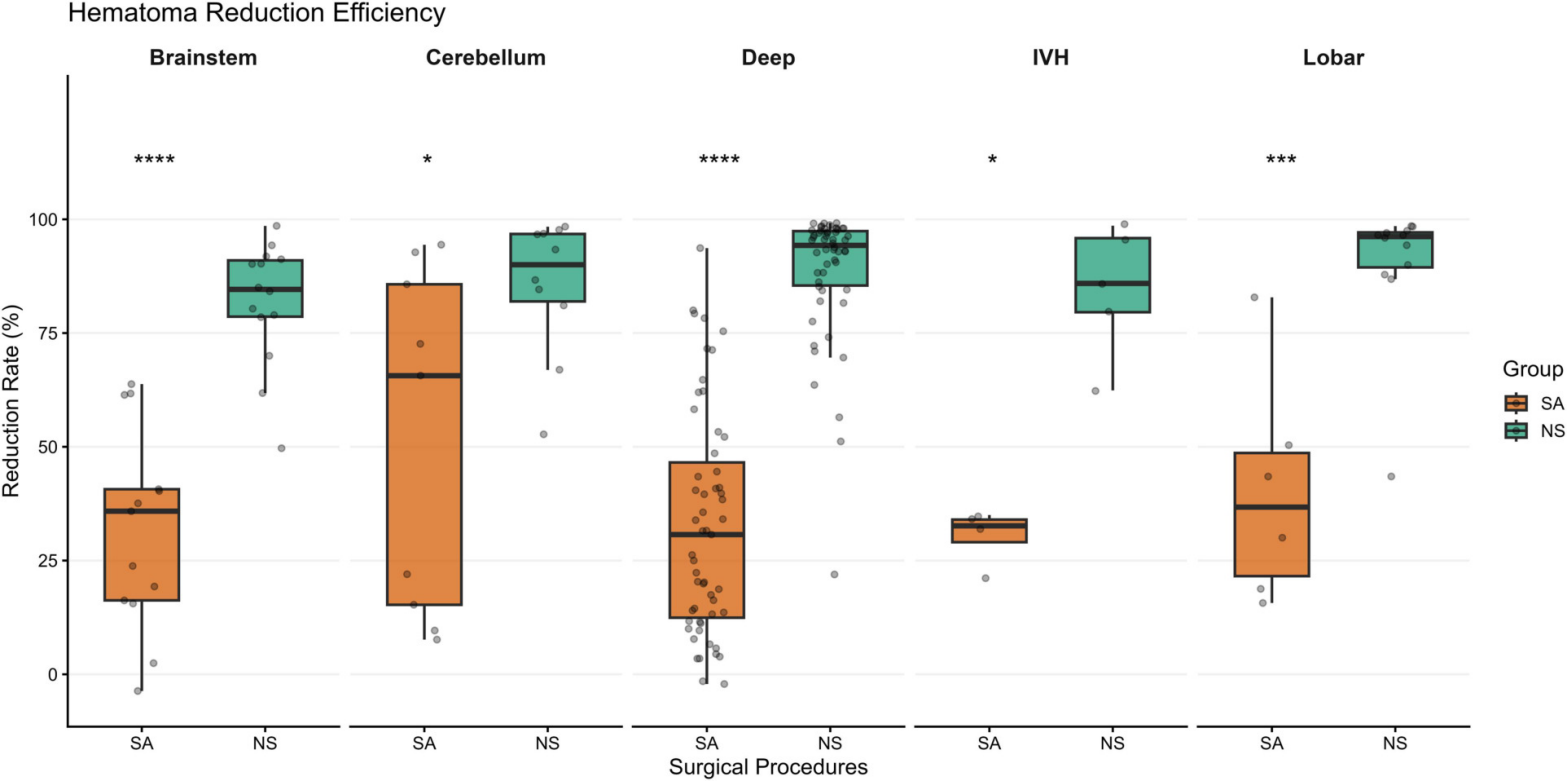
Comparison of Hematoma Volume Reduction. Boxplot illustrating the median percentage reduction in hematoma volume. NS was associated with significantly higher median hematoma reduction percentages than SA across all studied brain regions (****p < 0.001).

The NS approach was associated with a significantly higher median hematoma reduction rate compared to stereotactic aspiration. The overall median reduction rate was 92.9% [IQR: 82.0, 97.0] in the NS group, compared to 22.2% [IQR: 7.7, 43.5] in the SA group (*p* < 0.001; Figure 3).

**Figure 4:**
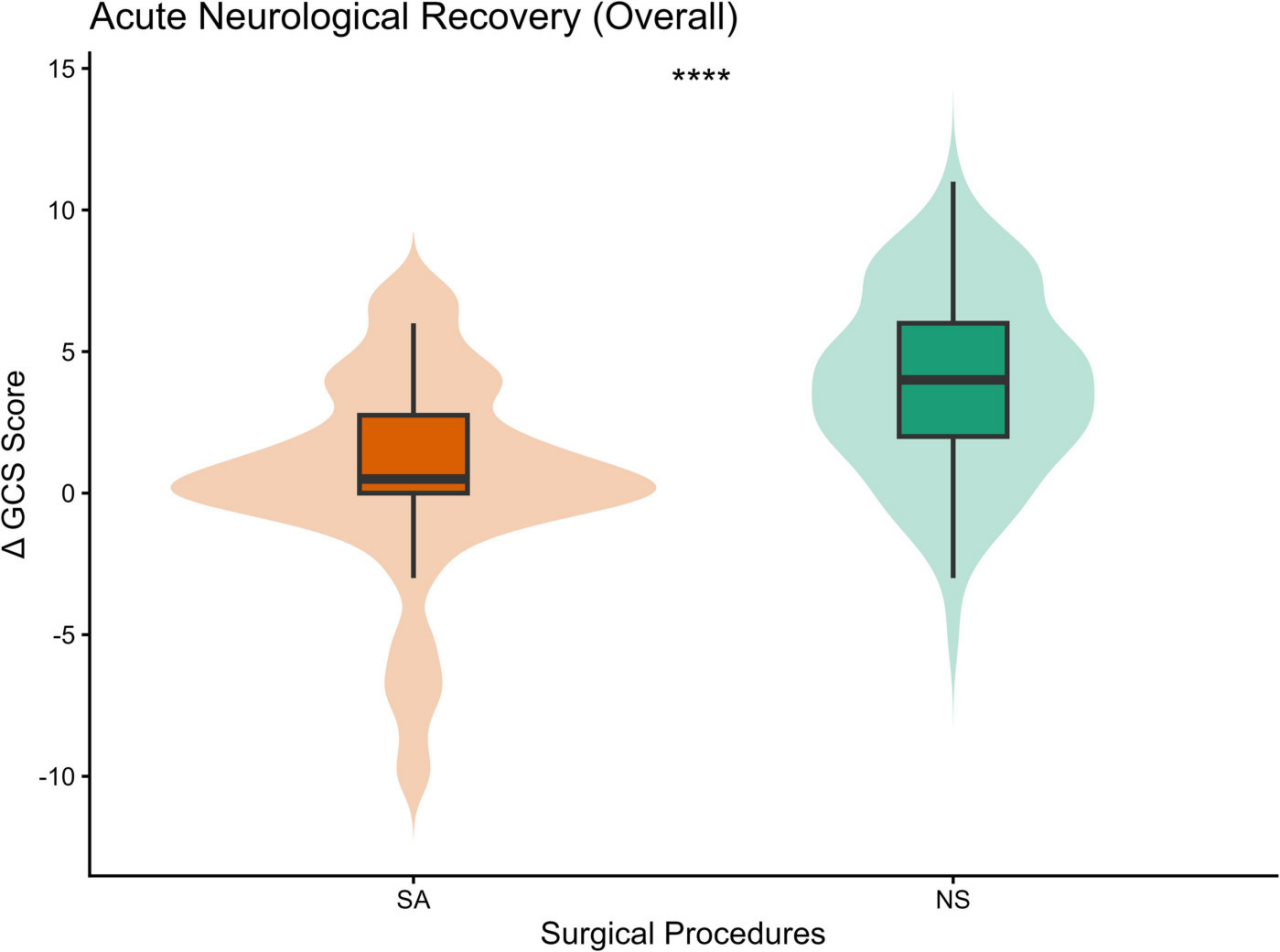
Acute Neurological Improvement (ΔGCS). Boxplot comparing the median change in GCS score at 24 h post-operatively. The NS group demonstrated a statistically significant median improvement of 4 points compared to 0.5 points in the SA group (****p < 0.001).

**Figure 5:**
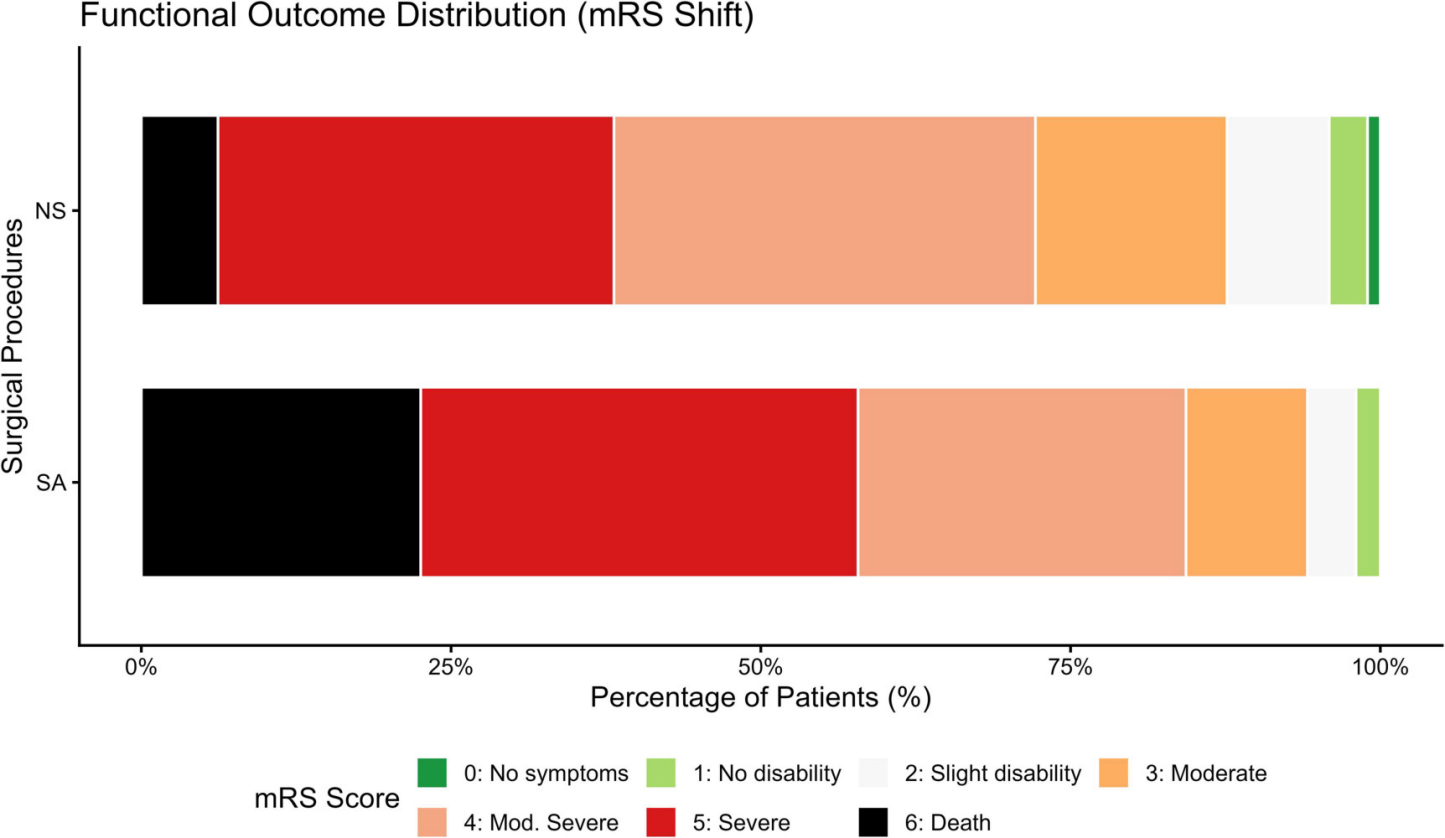
Distribution of mRS Scores at Discharge. A categorical shift analysis demonstrates a greater proportion of patients achieving functional independence (mRS 0–3) in the NS group. A score of 6 corresponds to 30-day mortality, highlighting the cohort's survival bias.

This higher degree of evacuation correlated with more pronounced acute neurological improvement. The median change in GCS score (ΔGCS) assessed at 24 h post-operatively was 4.00 [IQR: 2.00, 6.00] for NS patients, whereas SA patients demonstrated a median improvement of 0.50 [IQR: 0.00, 2.8] (*p* < 0.001; Figure 4). Furthermore, functional independence at discharge (mRS 0–3) was achieved by 27.8% of the NS group compared to 15.7% of the SA group (*p* = 0.040). The overall improvement in functional status in the NS group is illustrated in Figure 5.

### 3.3 Regional subgroup analysis

**Figure 6:**
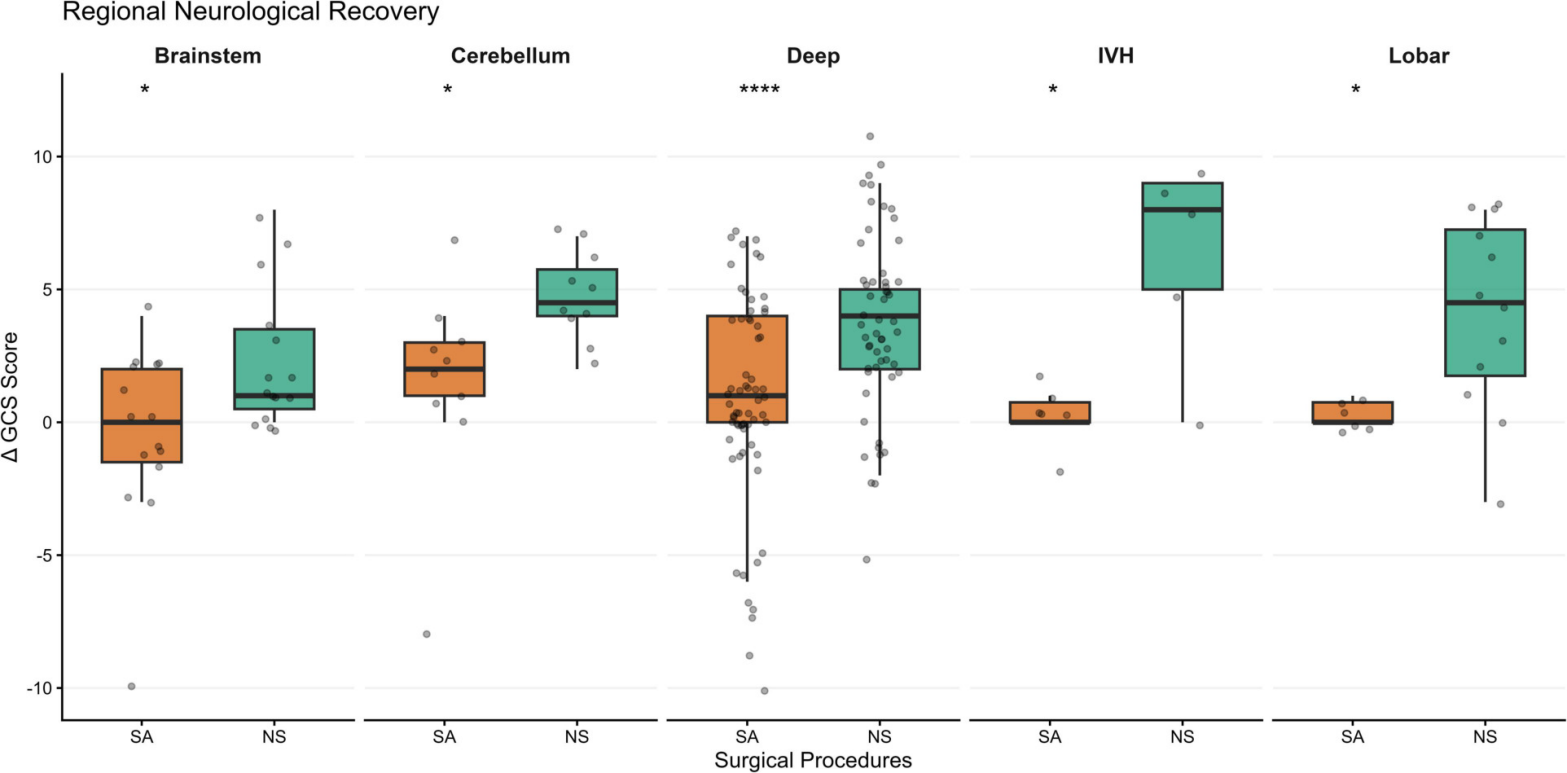
Regional Neurological Recovery. Stratified analysis showing median GCS score improvement (ΔGCS) across anatomical locations. The advantage associated with the NS remained consistent across deep, lobar, IVH, cerebellar and brainstem regions.

Subgroup analyses indicated that the radiological and neurological trends favoring NS were consistent across all studied locations. Significant differences favoring the NS approach were observed in the Deep-seated (Basal Ganglia/Thalamus), lobar, IVH, cerebellum, and brainstem subgroups for both radiological evacuation and neurological recovery (all *p* < 0.05; Figure 6).

### 3.4 Safety, predictors, and resource utilization

The incidence of symptomatic rebleeding was lower in the NS group compared to the SA group (7.2% vs. 24.5%; *p* < 0.001). The 30-day mortality rate was 9.3% in the NS group compared to 22.5% in the SA group (*p* = 0.012). Differences were observed in resource utilization, with the NS group exhibiting a median hospital stay of 18.0 days compared to 18.5 days for the SA group (*p* = 0.669). While the duration of mechanical ventilation was longer in the NS group (median 2.0 vs. 1.0 days; *p* < 0.001), this is largely influenced by survival bias within the cohort; the high early mortality rate in the SA group truncated the clinical course for non-survivors, whereas the higher survival rate in the NS group allowed for extended post-operative care and rehabilitation. Detailed outcomes by subgroup and surgical modality are summarized in Table 2.

**Table 2:** Clinical and radiological outcomes by surgical group.

Outcome measures	SA group (*n* = 102)	NS group (*n* = 97)	*p*-value
Radiological outcomes			
Hematoma reduction rate (%), med [IQR]	22.2 [7.7, 43.5]	92.9 [82.0, 97.0]	<0.001*
Basal ganglia (median)	25.0%	95.4%	<0.001*
Thalamus (median)	17.3%	92.9%	<0.001*
Brainstem (median)	23.8%	84.2%	<0.001*
Cerebellum (median)	43.8%	90.0%	0.015*
IVH (median)	26.5%	85.9%	0.004*
Lobar (median)	36.7%	96.2%	0.001*
Neurological outcomes			
GCS improvement (Δ), median [IQR]	0.50 [0.00, 2.80]	4.00 [2.00, 6.00]	<0.001*
Basal ganglia (median Δ)	+1.0	+5.0	<0.001*
Thalamus (median Δ)	+0.0	+2.0	0.036*
Brainstem (median Δ)	+0.0	+1.0	0.022*
Cerebellum (median Δ)	+2.0	+4.5	0.012*
IVH (median Δ)	+0.0	+8.0	0.039*
Lobar (median Δ)	+0.0	+4.5	0.020*
Good functional outcome (mRS 0–3), *n* (%)	16 (15.7%)	27 (27.8%)	0.040*
Safety and mortality			
Rebleeding, *n* (%)	25 (24.5%)	7 (7.2%)	<0.001*
30-day mortality, *n* (%)	23 (22.5%)	9 (9.3%)	0.012*
Resource utilization			
Hospital stay (days), median [IQR]^a^	18.5 [11.2, 26.0]	18.0 [14.0, 25.0]	0.669
Mechanical ventilation (days), med [IQR]^a^	1.0 [1.0, 1.0]	2.0 [1.0, 10.0]	<0.001*

Continuous variables are reported as median [interquartile range].

^a^ Higher resource utilization reflects survival bias; non-survivors in the SA group had truncated durations of care.

* Statistically significant (*p* < 0.05).

### 3.5 Multivariate predictors of functional outcome

To determine whether the observed surgical benefits were independent of baseline imbalances and clinical severity, a multivariate Firth's penalized logistic regression was performed. After adjusting for age, hypertension, pre-operative GCS, and hematoma volume, the NS approach remained a significant independent predictor of achieving a good functional outcome (mRS 0–3), with an adjusted odds ratio (OR) of 3.006 (95% CI 1.341–7.098; *p* = 0.007, Table 3).

**Table 3:** Multivariate predictors of good functional outcome (mRS 0–3).

Variable	Adjusted odds ratio (OR)	95% confidence interval	*p* value
Surgical group (NS vs. SA)	3.006	1.341–7.098	0.007*
Pre-op GCS score	1.386	1.229–1.585	<0.001*
Age	1.000	0.969–1.032	0.999
Hypertension (HTN)	0.638	0.286–1.389	0.259
Pre-op volume (mL)	0.997	0.981–1.012	0.720

* statistically significant.

### 3.6 Radiological evidence of decompression

**Figure 7:**
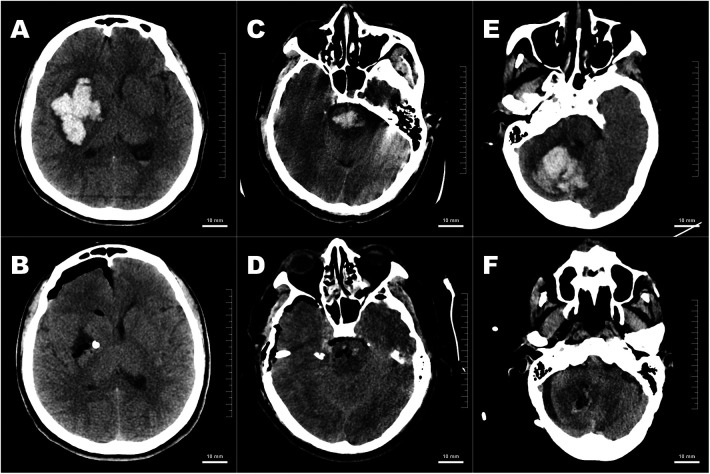
Pre- and postoperative axial CT images demonstrating hematoma evacuation across three hemorrhage locations. **(A,B)** Supratentorial (basal ganglia) hemorrhage: preoperative scan **(A)** shows a large hyperdense hematoma with a midline shift; postoperative scan **(B)** demonstrates near-complete clot evacuation and a reduction in the mass effect. **(C,D)** Brainstem (pontine) hemorrhage: Initial scan **(C)** reveals a dense central pontine hematoma; follow-up **(D)** shows a significant reduction in hematoma volume and an improved contour of adjacent structures. **(E,F)** Cerebellar hemorrhage: Preoperative image **(E)** indicating a space-occupying hematoma compressing the fourth ventricle; postoperative image **(F)** showing effective clot removal and decompression of posterior fossa contents.

Postoperative non-contrast CT imaging confirmed effective hematoma evacuation across the central intracranial locations studied. As illustrated in Figure 7, representative cases from the NS group demonstrate substantial reductions in hematoma volume and relief of secondary mass effect.

In the supratentorial basal ganglia case (A,B), the preoperative scan revealed a large hyperdense hematoma with significant midline shift; the postoperative scan demonstrates near complete clot evacuation and a marked reduction in mass effect. In the brainstem case (C,D), the initial scan revealed a dense central pontine hematoma; follow-up imaging shows a significant reduction in hematoma volume and improved contours of the adjacent brainstem structures. Similarly, the cerebellar case (E,F) involved a space-occupying hematoma compressing the fourth ventricle, which was effectively relieved following clot evacuation, restoring decompression of the posterior fossa contents, Figure 7.

## 4 Discussion

In this single-center retrospective cohort, NS was associated with greater early hematoma evacuation and acute neurological improvement than SA across multiple IPH locations. Our primary observation that NS achieved a significantly higher median hematoma reduction rate (92.90% vs. 22.20%, *p* < 0.001) and more pronounced neurological improvement (median ΔGCS 4.0 vs. 0.5 points, *p* < 0.001) is consistent with contemporary large-scale meta-analyses (18, 19) and recent cohort evidence demonstrating rapid recovery in large patient series (20). It is nonetheless essential to distinguish these radiological and neurological indicators as surrogate endpoints. While critical for immediate decompression, such markers may not directly or fully reflect the long-term, patient-centered functional trajectory (21, 22). To be considered a valid surrogate, a biomarker must lie within the pathophysiologic causal pathway and accurately capture the intervention's net effect on clinical outcome (21).

The degree of hematoma clearance is widely regarded as a critical determinant of secondary brain injury (12). The landmark MISTIE III trial established that reducing residual hematoma volume to 15 mL or less is a primary driver of functional improvement at 1 year (14, 23). Our results suggest that NS maintains a superior capacity to meet these evacuation targets consistently across diverse brain regions, an efficacy likely attributable to the fundamental advantage of direct visualization (24). Unlike the relatively “blind” approach of SA, which results in approximately 42% of patients failing to reach expected evacuation thresholds (23), NS enables meticulous, real-time evacuation and targeted identification and cauterization of active bleeding sources (16, 24). Advanced techniques, such as the Stereotactic Intracerebral Hemorrhage Underwater Blood Aspiration (SCUBA) method, have identified and controlled active bleeders in nearly half (48.9%) of cases, representing a crucial step toward preventing rebleeding and achieving comprehensive evacuation (24). This technical capability is further reflected in our observation of a significantly lower rebleeding rate in the NS group (7.2% vs. 24.5%, *p* < 0.001).

The clinical novelty of this study lies in its comprehensive analysis across different anatomical regions, providing comparative data across five distinct anatomical regions: Deep-seated (Basal Ganglia/Thalamus), Lobar, IVH, Brainstem, and Cerebellum. While the advantages of NS over SA have been reported in various supratentorial series (9, 19), our data suggest that the benefits of direct visualization and active hemostasis extend effectively to the challenging environments of the posterior fossa and brainstem. The management of deep-seated and infratentorial hemorrhages, particularly in the brainstem, has historically leaned toward conservative management. However, our findings align with recent evidence, such as that reported by Xu et al., which demonstrates that active surgical intervention is not only safe but provides superior functional outcomes (25). In their cohort, patients treated with robotic-assisted stereotactic aspiration showed significantly higher Glasgow Outcome Scale (GOS) scores compared to those managed conservatively. Our data extends this observation: when comparing NS against SA, we observed that patients with brainstem hemorrhages treated via NS demonstrated a median GCS improvement of +1.0 compared to +0.0 in the SA group (*p* = 0.022), suggesting the potential for active, minimally invasive intervention to improve neurological recovery in these high-risk cohorts (13, 26).

Regarding cast Intraventricular Hemorrhage (IVH), our observations support those of recent comparative studies by Di Rienzo et al., which indicate that endoscope-assisted evacuation is superior to External Ventricular Drainage (EVD) alone. Traditional EVD management is frequently limited by catheter obstruction and protracted CSF clearance times (27). In contrast, our NS approach facilitated faster hematoma clearance and reduced ICU stays. For cerebellar hemorrhage, our results support emerging evidence that NS offers a safe and secure alternative to open craniotomy in the confined posterior fossa, resulting in lower infection and cerebrospinal fluid leak rates (28, 29).

An essential observation in our analysis was the “survival paradox” regarding resource utilization. While the NS group appeared to have similar median hospital stays (18.0 vs. 18.5 days) and slightly longer ventilation durations (2.0 vs. 1.0 days), these metrics must be interpreted in light of the disparate 30-day mortality rates. The significantly higher early mortality in the SA group (22.5%) truncated the clinical course for nearly one quarter of the cohort, naturally lowering the group's average resource use (9). Conversely, 90.7% of patients in the NS group survived to undergo extended post-operative care and rehabilitation. After adjusting for baseline imbalances in age, hypertension, and initial GCS using Firth's penalized likelihood regression, the surgical approach remained an independent predictor of achieving functional independence at discharge (OR: 3.006; 95% CI: 1.341–7.098), a finding consistent with recent comparative prognosis analyses (17). Also aligns with the ENRICH trial, which found that minimally invasive intervention significantly decreased ICU and hospital lengths of stay among survivors (30).

Despite the radiological and early clinical advantages of NS, we acknowledge the procedural benefits of SA. SA typically involves shorter operative times and minimal intraoperative blood loss, making it a viable alternative for patients with severe systemic comorbidities who may not tolerate extended general anesthesia (11, 19). The principal limitations of our study include its single-center, retrospective design and relatively small sample sizes for specific anatomical subgroups (brainstem or cerebellum). Furthermore, as identified during the screening process, several patients were excluded due to a loss of long-term follow-up or incomplete medical and radiological records. Our analysis was also limited by a focus on hypertension and diabetes mellitus as primary comorbidities because data were lacking regarding other critical prognostic factors, such as anticoagulation status, hematoma expansion, and high-risk radiological features (e.g., the spot sign or hypodensity sign). Nevertheless, the consistency of our findings with international datasets supports the generalizability of our observations. Future prospective randomized trials, such as the long-term results of ENRICH, which reported a 98.1% posterior probability of surgery's superiority for lobar hemorrhages, will be critical to determine whether these early surrogate benefits translate into improved long-term functional independence at 6–12 months (30).

## 5 Conclusion

This retrospective study of IPH across different anatomical locations demonstrates that NS is associated with significantly higher hematoma evacuation efficiency and more pronounced early neurological recovery across a diverse range of IPH locations compared to SA. By facilitating direct visualization and active hemostasis, the endoscopic approach achieved greater radiological clearance and was associated with a notable reduction in 30-day mortality and symptomatic rebleeding within this cohort, with results appearing robust across both supratentorial and infratentorial compartments. While the observed benefits in radiological clearance and acute GCS improvement serve as critical surrogate endpoints for successful decompression, the ultimate transition of these technical benefits into improved long term functional quality of life remains to be confirmed through prospective, multicenter randomized controlled trials; nevertheless, our findings support NS as a potentially versatile and practical minimally invasive option for rapid clot evacuation in patients presenting with IPH in different anatomical locations.

## Data Availability

The original contributions presented in the study are included in the article/Supplementary Material, further inquiries can be directed to the corresponding authors.

## References

[1] De Oliveira Manoel AL. Surgery for spontaneous intracerebral hemorrhage. Crit Care. (2020) 24:45. 10.1186/s13054-020-2749-2 32033578 PMC7006102

[2] Cai Q Zhang H Zhao D Yang Z Hu K Wang L Analysis of three surgical treatments for spontaneous supratentorial intracerebral hemorrhage. Medicine. (2017) 96:e8435. 10.1097/MD.0000000000008435 29069046 PMC5671879

[3] Ma H Peng W Xu S Liang X Zhao R Lv M Advancements of endoscopic surgery for spontaneous intracerebral hemorrhage. World Neurosurg. (2025) 193:160–70. 10.1016/j.wneu.2024.10.107 39491620

[4] Cai Q Guo Q Li Z Wang W Zhang W Ji B Minimally invasive evacuation of spontaneous supratentorial intracerebral hemorrhage by transcranial neuroendoscopic approach. Neuropsychiatr Dis Treat. (2019) 15:919–25. 10.2147/NDT.S195275 31043783 PMC6469739

[5] Li L Li Z Li Y Su R Wang B Gao L Surgical evacuation of spontaneous cerebellar hemorrhage: comparison of safety and efficacy of suboccipital craniotomy, stereotactic aspiration, and thrombolysis and endoscopic surgery. World Neurosurg. (2018) 117:e90–8. 10.1016/j.wneu.2018.05.170 29864571

[6] Li L Liu H Luo J Tan Z Gao J Wang P Comparison of long-term outcomes of endoscopic and minimally invasive catheter evacuation for the treatment of spontaneous cerebellar hemorrhage. Transl Stroke Res. (2021) 12:57–64. 10.1007/s12975-020-00827-8 32623579 PMC7803713

[7] Wei M Chen Q Yang X Zhu X Tian X Tong Q 3-Dimensional technology-assisted minimally invasive surgery for the treatment of primary brainstem hemorrhage: a prospective cohort study. World Neurosurg. (2025) 194:123487. 10.1016/j.wneu.2024.11.070 39579926

[8] Hedaoo K Sinha M Chauhan BPS Bajaj J Ratre S Swamy MN Neuroendoscopy training. Asian J Neurosurg. (2025) 20:1–9. 10.1055/s-0044-1791713 40041584 PMC11875708

[9] Tahara S Hattori Y Aso S Uda K Kumazawa R Matsui H Outcomes after endoscopic evacuation versus evacuation using craniotomy or stereotactic aspiration for spontaneous intracerebral hemorrhage: analysis using a Japanese nationwide database. Neurocrit Care. (2023) 38:667–75. 10.1007/s12028-022-01634-9 36348138

[10] Xu X Zheng Y Chen X Li F Zhang H Ge X. Comparison of endoscopic evacuation, stereotactic aspiration and craniotomy for the treatment of supratentorial hypertensive intracerebral haemorrhage: study protocol for a randomised controlled trial. Trials. (2017) 18:296. 10.1186/s13063-017-2041-1 28659171 PMC5490150

[11] Xu X Zhang H Zhang J Luo M Wang Q Zhao Y Minimally invasive surgeries for spontaneous hypertensive intracerebral hemorrhage (MISICH): a multicenter randomized controlled trial. BMC Med. (2024) 22:244. 10.1186/s12916-024-03468-y 38867192 PMC11170771

[12] Ray PS. Surgery for spontaneous intracerebral hemorrhage: current concept. Indian J Neurosurg. (2021) 10:1–5. 10.1055/s-0041-1726865

[13] Wang G Chen X Meng L Liu Y Dai Y Wang W. The application effect of craniotomy through transsylvian rolandic point-insular approach on hypertensive intracerebral hemorrhage in posterior basal ganglia. Behav Neurol. (2023) 2023:1–11. 10.1155/2023/2266691 PMC10699897 38074419

[14] Hanley DF Thompson RE Rosenblum M Yenokyan G Lane K McBee N Minimally invasive surgery with thrombolysis in intracerebral haemorrhage evacuation (MISTIE III): a randomised, controlled, open-label phase 3 trial with blinded endpoint. Lancet. (2019) 393:1021–32. 10.1016/S0140-6736(19)30195-3 30739747 PMC6894906

[15] Mendelow AD Gregson BA Fernandes HM Murray GD Teasdale GM Hope DT Early surgery versus initial conservative treatment in patients with spontaneous supratentorial intracerebral haematomas in the international surgical trial in intracerebral haemorrhage (STICH): a randomised trial. Lancet. (2005) 365:387–97. 10.1016/S0140-6736(05)17826-X 15680453

[16] Mascitelli JR Bain MD Kellner CP. Minimally invasive intracerebral hematoma evacuation: a difficult road, but a bright future. J Neurointerv Surg. (2025) 17:788–9. 10.1136/jnis-2025-023941 40602999

[17] Guo T Zhu Q Zhu C Zhao Z Chai H Pan X Comparative analysis of neurological function and prognosis after stereotactic aspiration and neuroendoscopic surgery for hypertensive intracerebral hemorrhage. Ann Ital Chir. (2025) 96:514–22. 10.62713/aic.3950 40234220

[18] Sun S Huang X Fei X Gong K Ye F Gao H. Neuroendoscopic surgery versus stereotactic aspiration in the treatment of supratentorial intracerebral hemorrhage: a meta-analysis. World Neurosurg. (2024) 187:e585–97. 10.1016/j.wneu.2024.04.132 38679374

[19] Yang L Yang M He M Zhou X Zhou Z. Endoscopic surgery versus stereotactic aspiration in spontaneous intracerebral hemorrhage treatment: a systematic review and meta-analysis. World Neurosurg. (2024) 184:202–12. 10.1016/j.wneu.2024.01.162 38316176

[20] Shafiq Z Cao F Lu M Li Z Song P Zhou L Neuroendoscopy for acute severe neurological conditions: high hematoma clearance and rapid recovery in 815 patients – cohort study. Int J Surg. (2025) 111:10-1097. 10.1097/JS9.0000000000004491 PMC13105529 41376305

[21] Fleming TR Powers JH. Biomarkers and surrogate endpoints in clinical trials. Stat Med. (2012) 31:2973–84. 10.1002/sim.5403 22711298 PMC3551627

[22] Chalos V van der Ende NAM Lingsma HF Mulder MJHL Venema E Dijkland SA National institutes of health stroke scale: an alternative primary outcome measure for trials of acute treatment for ischemic stroke. Stroke. (2020) 51:282–90. 10.1161/STROKEAHA.119.026791 31795895 PMC6924951

[23] Luo X Song K Zhuo L Lin F Gao Z He Q Analysis of associated factors affecting hematoma evacuation rates in spontaneous intracerebral hemorrhage with stereotactic aspiration combined with catheter drainage. Sci Rep. (2025) 15:17759. 10.1038/s41598-025-01754-0 40404752 PMC12098909

[24] Kellner CP Chartrain AG Nistal DA Scaggiante J Hom D Ghatan S The stereotactic intracerebral hemorrhage underwater blood aspiration (SCUBA) technique for minimally invasive endoscopic intracerebral hemorrhage evacuation. J Neurointerv Surg. (2018) 10:771–6. 10.1136/neurintsurg-2017-013719 29572265 PMC6278654

[25] Xu C He W Yi T Zhang H Xu J Ma J. Robotic frameless stereotactic aspiration with thrombolysis for primary pontine hemorrhage: a therapeutic evaluation of a retrospective cohort study. J Neurol Surg A Cent Eur Neurosurg. (2025) 86:111–9. 10.1055/a-2235-5453 38151032

[26] Shafiq Z Zhou L Jiang X Li Z Song P Zhang S Triple sheath neuroendoscopic combination technique for managing complete intraventricular hemorrhage casting in patients with cerebral hemorrhage. Front Neurol. (2025) 16:1554187. 10.3389/fneur.2025.1554187 40534739 PMC12173931

[27] Di Rienzo A Colasanti R Esposito D Della Costanza M Carrassi E Capece M Endoscope-assisted microsurgical evacuation versus external ventricular drainage for the treatment of cast intraventricular hemorrhage: results of a comparative series. Neurosurg Rev. (2020) 43:695–708. 10.1007/s10143-019-01110-7 31069562

[28] Atsumi H Baba T Sunaga A Sakakibara Y Nonaka Y Sorimachi T Neuroendoscopic evacuation for spontaneous cerebellar hemorrhage is a safe and secure approach and may become a mainstream technique. Neurol Med Chir. (2019) 59:423–9. 10.2176/nmc.oa.2019-0108 PMC6867934 31582641

[29] Monteiro GA Marinheiro G Mutarelli A Araújo B Cavalcante-Neto JF Batista S Efficacy and safety of neuroendoscopy surgery versus craniotomy for supratentorial intracerebral hemorrhage: an updated meta-analysis of randomized controlled trials. Neurosurg Rev. (2024) 47:255. 10.1007/s10143-024-02492-z 38833192

[30] Pradilla G Ratcliff JJ Hall AJ Saville BR Allen JW Paulon G Trial of early minimally invasive removal of intracerebral hemorrhage. N Engl J Med. (2024) 390:1277–89. 10.1056/NEJMoa2308440 38598795

